# Two cases of lead poisoning associated with occupational exposure presented as intestinal obstruction: a case report

**DOI:** 10.1093/jscr/rjac281

**Published:** 2022-06-22

**Authors:** Paran Tanwar, Pradeep Goyal, Sourabh Trivedi, Jahnavi Kolli

**Affiliations:** Department of Surgery, Maharishi Markandeshwar Medical College & Hospital, Solan, Himachal Pradesh, India; Department of Surgery, Maharishi Markandeshwar Medical College & Hospital, Solan, Himachal Pradesh, India; Department of Surgery, Maharishi Markandeshwar Medical College & Hospital, Solan, Himachal Pradesh, India; Department of Surgery, Maharishi Markandeshwar Medical College & Hospital, Solan, Himachal Pradesh, India

## Abstract

Lead exposure in India is common in industries that may present with nonspecific signs and symptoms or symptoms of toxicity, depending on the amount of lead absorbed. We are presenting two case reports of occupational lead exposure in a lead-based battery manufacturing unit, which were presented as intestinal obstruction. Both the patients aged 28 and 24-year old presented with complaints of pain in the abdomen with blood lead levels of 61.1 and 85 μg/dl, respectively. The diagnosis was made clinically before any other radiological investigation or intervention. Both the patients were treated conservatively with D-Penicillamine, following which, both the patients improved symptomatically and the blood lead level also decreased. The importance of a detailed history of occupation & clinically diagnosing such patient will avoid unnecessary investigation and intervention. This will help to reach the correct diagnosis in such cases.

## INTRODUCTION

Lead exposure in India is common in industries like batteries, pigments or paint, paper-hanging, lead and ore mining, smelting and refining, welding, soldering, ammunitions, car radiators, cable and wires, construction and demolition, some cosmetics, ceramics with lead glazes, plumbing and tin cans [1–3]. Lead poisoning may present with nonspecific signs and symptoms or severe symptoms of toxicity, depending on the amount of lead absorbed. In keeping with current practice by the US Centers for Disease Control and Prevention (CDC), ‘adult lead toxicity’ is defined as mean BLL ≥ 10 μg/dl. ‘Adult lead poisoning’ is defined as adult lead toxicity along with symptoms or signs [1]. We are discussing two case reports of occupational lead exposure in a lead-based battery manufacturing unit, which were presented as intestinal obstruction.

## CASE 1

**Figure 1:**
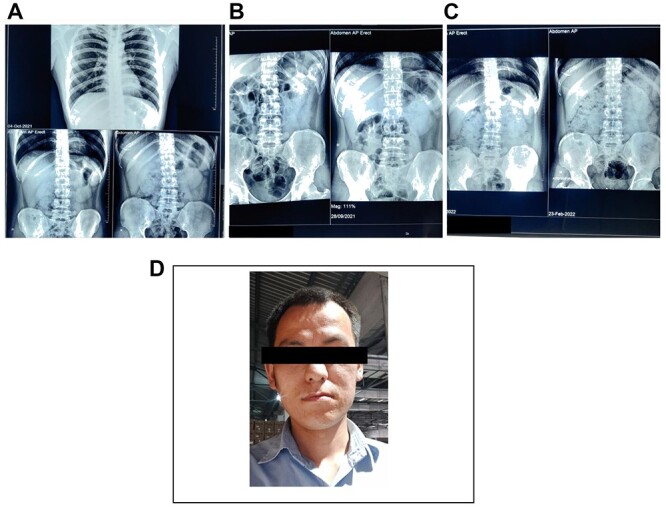
(**A**) and (**B**) Showing radiograph abdomen before the treatment. (**C**) Showing radiograph abdomen after the treatment. (**D**) Case 1—a 28-year-old male who worked as an engineer at lead battery manufacturing factory at Nalagarh (Himachal Pradesh) for last 6 months.

A 28-year-old male presented to surgery out-patient department with a chief complaint of pain in lower abdomen for 2 weeks (Fig. 1D). The pain was localized to the supra-pubic region, & associated with nausea. The patient also complains of absence of flatulence and stool for 2–3 days. Hemoglobin was 9.7 gm/dl (Table 1). Contrast enhanced computed tomography (CECT) abdomen showed hepatomegaly with diffuse hepatic steatosis. On detailed history, the patient revealed his occupational history of working as an engineer in a factory producing lead batteries. To rule out lead poisoning as the cause, serum lead levels were investigated, which turned out to be 61.1 μg/dl. The patient’s symptoms, examination, lab investigations, radiological investigations and serum lead levels helped reach us to the final diagnosis of lead poisoning. The patient was managed conservatively. D-Penicillamine 500 mg, orally for 15 days, along with laxatives and analgesics, were administered. The patient was kept on follow-up advice after 2 weeks. The blood lead levels came out as 37.969 μg/dl after the first cycle of chelation therapy, and the patient improved symptomatically. The patient was advised to continue the treatment and was called for a follow-up after 2 weeks. On second follow up, the blood lead levels increased to 41.026 μg/dl (Table 2) & hemoglobin levels decreased to 14.4 gm/dl.

**Table 1:** Showing blood lead levels after before and each cycle of treatment cycle

	**Before treatment**	**After first treatment cycle**	**After second treatment cycle**
Blood lead levels	61.1 μg/dl	37.969 μg/dl	41.026 μg/dl

**Table 2:** Complete blood count of case 1

	**Before treatment**	**After second treatment cycle**
Hemoglobin level	9.7 gm/dl	14.4 gm/dl
Red blood cell count	3.38 millions/mm^3^	5.18 millions/mm^3^
Packed cell volume	30.7%	43.9%
Mean corpuscular hemoglobin	28.7 pg	27.8 pg

The patient improved symptomatically, but the blood lead levels increased due to continued occupational exposure to lead.

## CASE 2

**Figure 2:**
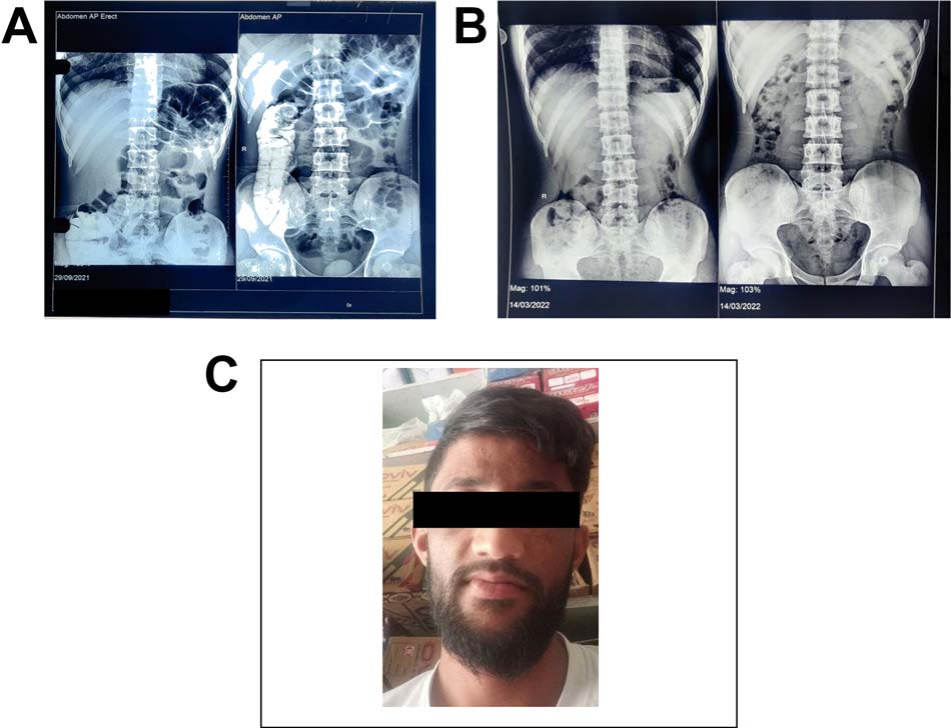
(**A**) Showing radiograph abdomen before the treatment. (**B**) Showing radiograph abdomen after the treatment. (**C**) Case 2—a 24-year-old male who worked as a laborer in a lead battery manufacturing factory in Nalagarh (Himachal Pradesh) for 5 years.

A 24-year-old male presented to our surgery out-patient department with a chief complaint of pain in abdomen for 5 days and nausea associated with 4–5 episodes of vomiting (Fig. 2C). The patient also complained about alternated bowel habits. Patient had similar complaints in 2018 and 2020, for which he received treatment in the form of laxatives and analgesics and improved. Radiograph abdomen (Erect) revealed dilatation of small bowel and large bowel in caliber suggesting bowel obstruction (Fig. 2). Hemoglobin was 8.5 g/dl (Table 3). CECT showed that dilated air distended small bowel loops and the entire colon with the moderate fecal matter within the right colon without transition point suggesting ileus. On detailed history, the patient revealed his occupational history of working as a laborer for 5 years in factory manufacturing lead batteries. To rule out lead poisoning as the cause, serum lead levels were evaluated, which turned out to be 85 μg/dl (Table 4). Patients symptoms, history, examination followed by investigation and serum lead levels helped us to reach the final diagnosis of Lead poisoning with paralytic ileus. The patient was managed. D-Penicillamine 500 mg, orally for 15 days, along with laxatives and analgesics, were administered. The patient improved symptomatically and has been kept for follow-up advice after 2 weeks. Serum Lead levels were 46.668 μg/dl, and the patient improved symptomatically. The patient was advised to continue the treatment and was called for a follow-up after two weeks. The patient improved symptomatically, but the blood lead levels came out 18 μg/dl. The patient was counseled about the hazards of occupational exposure, after which he discontinued the job at the factory.

**Table 3:** Blood lead levels after before and each cycle of treatment cycle

	Before treatment	After first treatment cycle	After second treatment cycle
Blood lead levels	85 μg/dl	46.668 μg/dl	18 μg/dl

**Table 4:** Complete blood count of case 2

	**Before treatment**	**After second treatment cycle**
Hemoglobin level	8.5 gm/dl	14.2 gm/dl
Red blood cell count	3.08 millions/mm^3^	4.84 millions/mm^3^
Packed cell volume	28.4%	43%
Mean corpuscular hemoglobin	27.6 pg	29.3 pg

## DISCUSSION

The extensive use of lead in India is turning out to be a hazard to the environment and human health [4]. Many studies and case reports indicate that lead poisoning is not uncommon in India [5–7]. The most common reason for lead exposure in adults is workplaces [2, 3]. Workplace exposure to lead can occur in numerous settings, including work that involves batteries, pigments or paint, paper-hanging, etc. [3]. Ingestion of lead in the human body can severely impact physiologic functions and the organ system [3–11]. Some of the toxic effects of lead (such as lead colic and anemia) are reversible if lead poisoning is identified early and managed effectively. However, high lead levels or moderate levels over long periods can result in irreversible damage to the central and peripheral nervous systems, kidneys and other organs [3, 9, 10]. The symptoms of lead toxicity usually appear at a blood lead level of 25–50 μg/dl in children and 40–60 μg/dl in adults [12]. There are many biochemical mechanisms by which the toxicity of lead poisoning can occur. Aminolevulinic acid dehydratase (ALAD) and ferrochelatase are porphyrin synthesis enzymes inhibited by lead toxicity. This inhibition leads to anemia [13]. Decreased pre-ganglionic acetylcholine release and intestinal Na/K-ATPase inhibition results in water influx abnormality, which leads to intestinal obstruction and constipation [14]. Anemia and gastrointestinal symptoms are prevalent in lead toxicity, but these symptoms are very generalized and may often mislead in the absence of a history of exposure. The most common symptom is abdominal pain, whereas signs and symptoms suggestive of toxic dilation of colon or bowel obstruction are uncommon [4]. In both the cases that we report, intestinal obstruction was diagnosed clinically, supplemented and confirmed radiologically, but the cause was unidentified. The possibility of lead exposure has been kept in mind while managing these cases. Lead poisoning has only one definitive test for diagnosis, i.e. measurement of blood lead levels. WHO defines whole blood lead concentration > 30 μg/dl adult as indicative of significant exposure. Lead concentration > 60 μg/dl requires chelation therapy [13]. Blood lead levels was very high in both cases, i.e. 85 and 61.1 μg/dl, respectively, and both required chelation therapy. Both patients were managed conservatively in surgery department in consultation with physician.

## CONFLICT OF INTEREST STATEMENT

None declared.

## FUNDING

None declared.
